# American Medical Productions and the English Critics

**Published:** 1851-11

**Authors:** 


☞ We take pleasure in transferring the following appropriate and correct remarks, from the *New Orleans Medical and Surgical Journal*, to our Journal.

"*American Medical Productions and the English Critics.*—In the April number for 1851, of the *British and Foreign Medical Review*, is contained an elaborate review and criticism of Professor Drake's great work on the Principal Diseases of North America. This review, taken as a whole, is decidedly complimentary of the work, and it is so rare for our transatlantic ancestors to speak well of any *American* literary productions, that we cannot withhold the expression of both our surprise and satisfaction at this change of sentiments.

"In medical literature, we must, to be honest, plead guilty to the serious charge laid at our doors, in the following quotation from the above work; but Dr. Drake has produced a book which will, in all probability, give a new direction to the medical literature of this country, and tend to reprove, if not repress, the bitter, but just sarcasm levelled at the Profession of America. The Reviewer concludes his notice of Dr. D.'s book in these words:

'We sincerely hope, however, that there is no danger of so useful a work, (alluding to the author's doubts about the success of his book,) not meeting with sufficient encouragement in America, to induce its veteran author to complete it without delay. We have had occasion to observe of late upon the dearth of medical works in that country, having pretensions to originality and research; native talent seeming to be wholly expended in dishing up and garnishing the productions of European pens, and it will, indeed, be melancholy to find an author, who has had the courage to produce a work of this magnitude, and one which would do honor to any country, left the sole consolation of a posthumous reputation.'

"The foregoing strictures are well merited, as applied to book-makers; but when we come to speak of the character and spirit of the *original* articles published in *American Medical Journals*, we cannot see that we are much behind, in practical knowledge and originality of thought, our medical brethren on the other side of the Atlantic. The *cui bono* is the first thought with the American physician; he always takes the most direct route to gain an end, in despite of obstacles that might baffle and discomfit the more cautious and circumspect physician. Feats in surgery, obstetrics, and practical medicine, are being almost daily achieved in the wild woods of our almost boundless Union, which, if performed by a Londoner, might induce Her Majesty to confer upon him the title of Baronet."

